# Supplementary material for: ATAC-Seq and RNA-Seq Integration Reveals Chromatin Accessibility and Transcriptional Dynamics During Fruit Color Development in Mulberry
Source: Int J Mol Sci. 2026 Jan 1;27(1):456. doi: 10.3390/ijms27010456 (PMC12786836; doi:10.3390/ijms27010456)
Supplement: Supplementary file 1 [file ijms-27-00456-s001.zip › Supplementary figure.pdf]

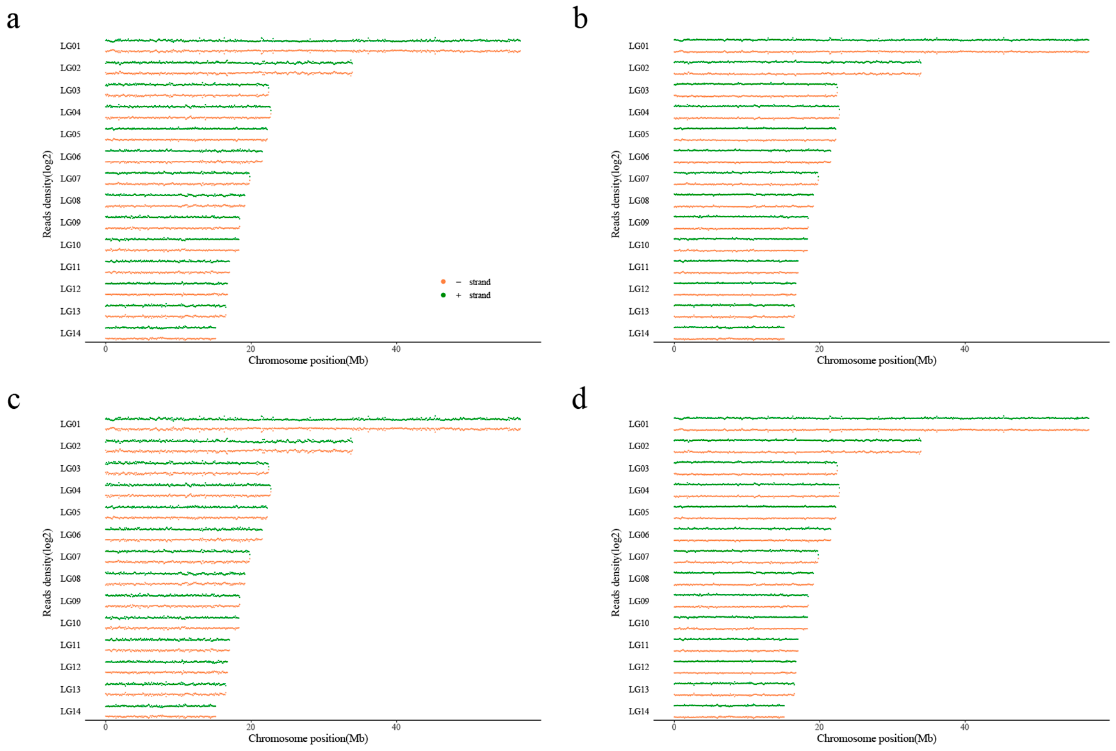

Figure S1: The reads coverage of ATAC-seq along chromosomes of S1(a and b) and S3 (c and d) samples.

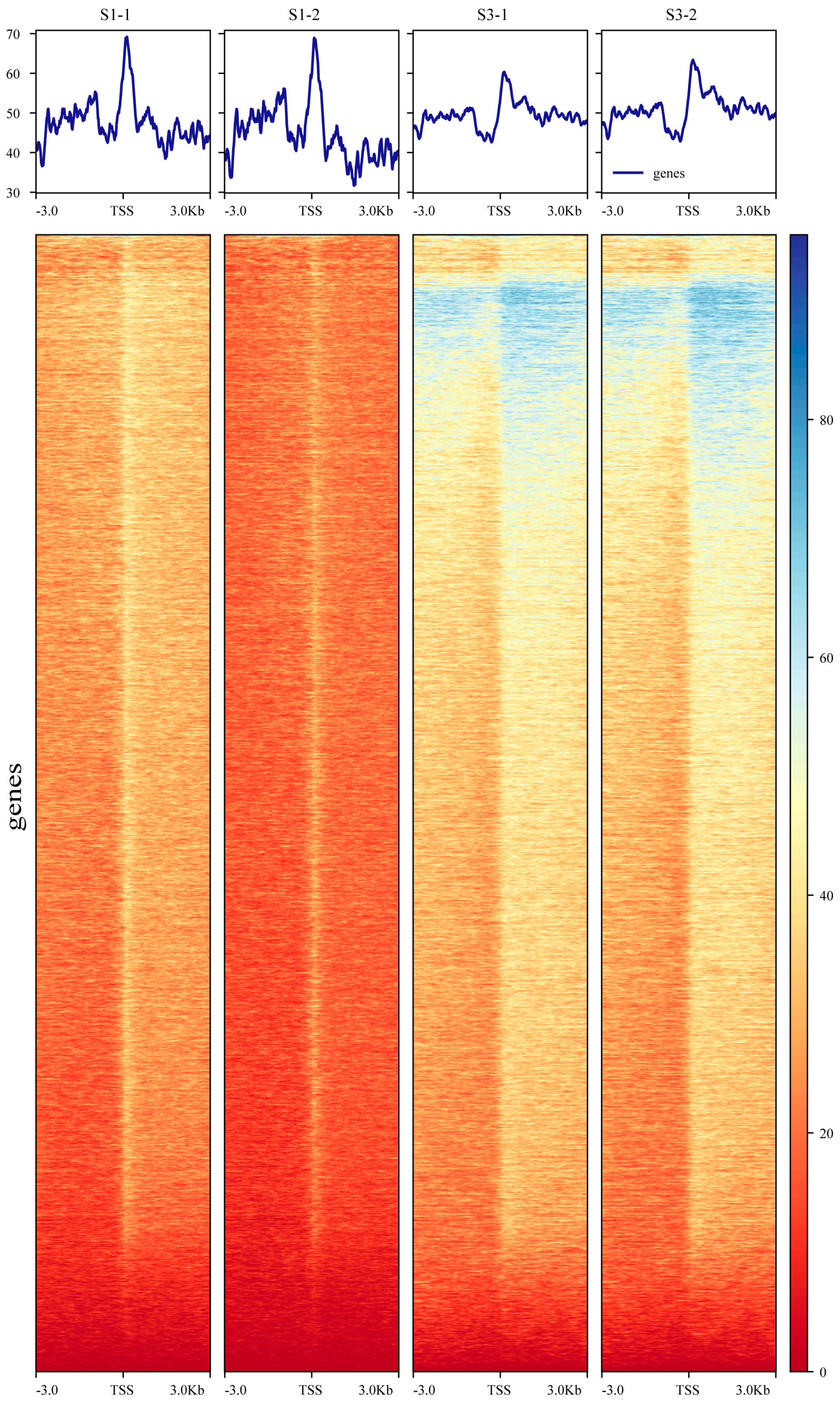

Figure S2: TSS enrichment of ATAC-seq reads.

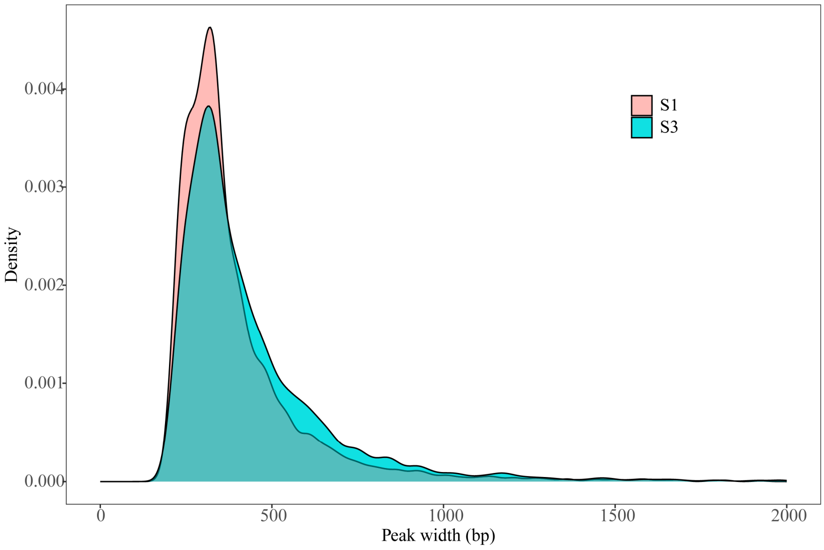

Figure S3: The mean width of high-confidence peaks.

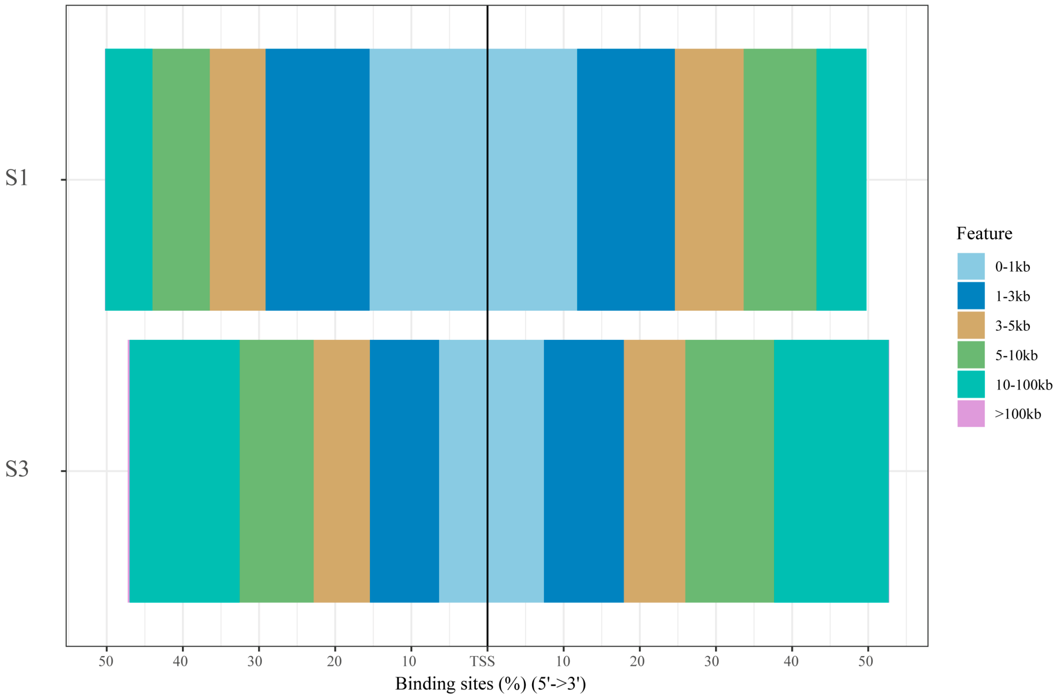

Figure S4: Distribution of transcription factor-binding loci n relative to TSS.

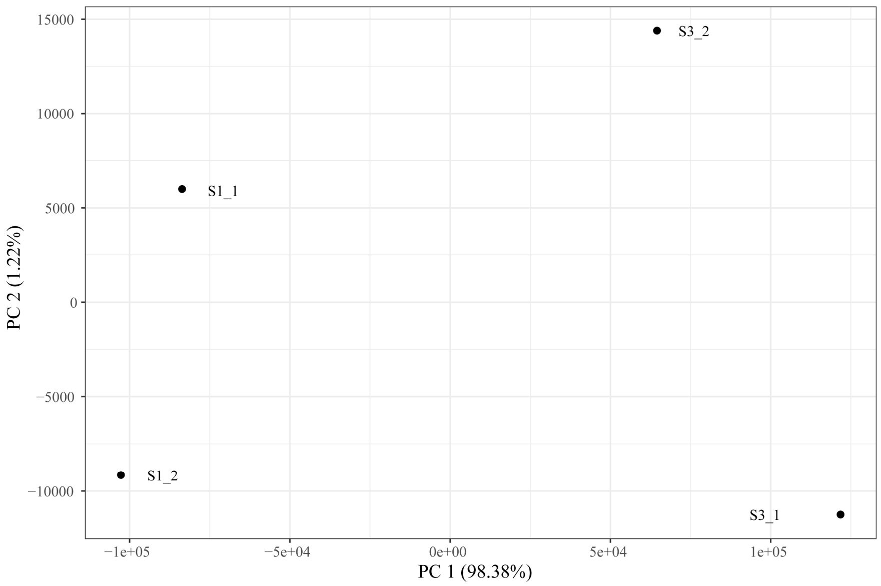

Figure S5: PCA analysis of four ATAC-seq data.

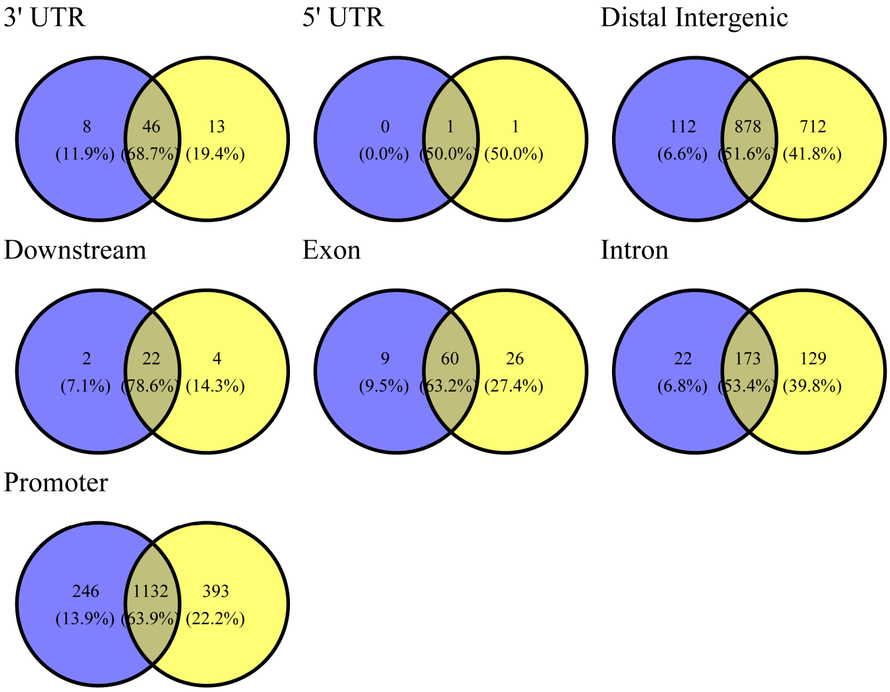

Figure S6: Venn diagram showing the shared THS-associated genes between two ATAC-seq data sets. Purple and yellow represent S1 and S3 samples, respectively.

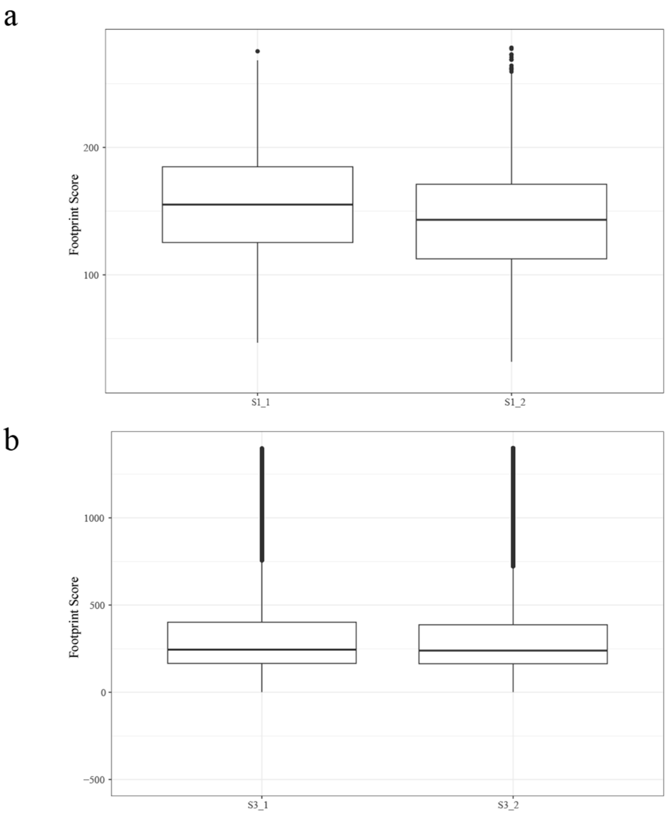

Figure S7: boxplots showing footprint scores between biological replicates of S1 (a) and S3 (b).

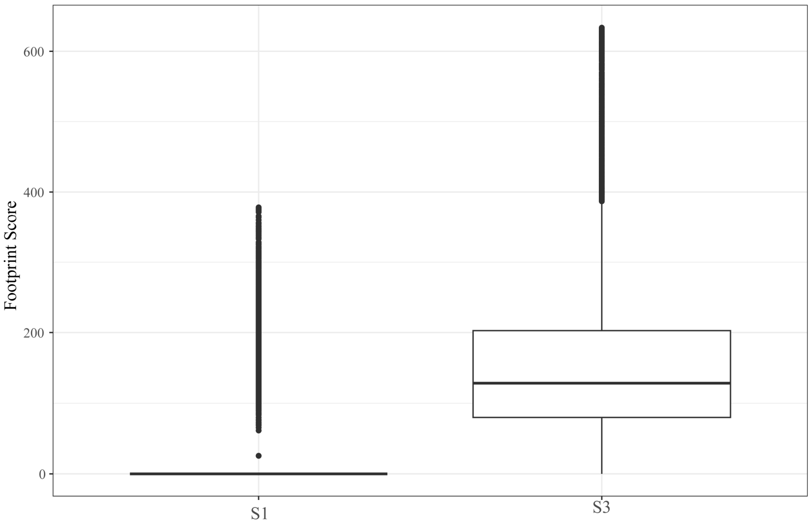

Figure S8: Footprint scores between the two developmental stages.

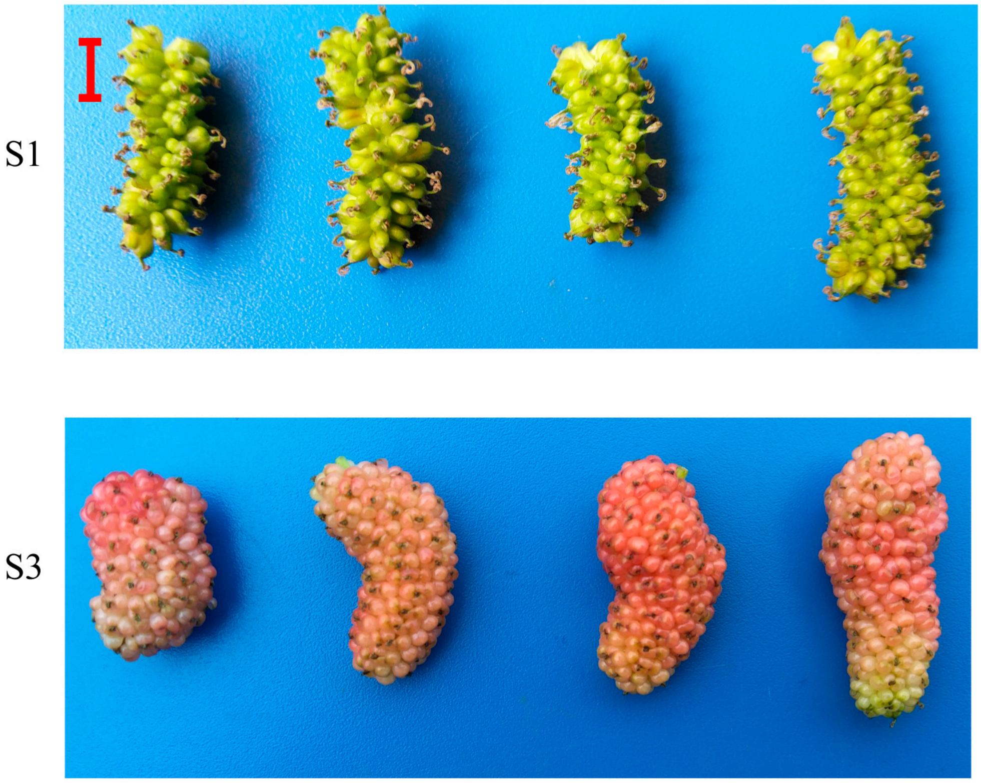

Figure S9: The appearance of mulberry fruits with S1 and S3 stage.

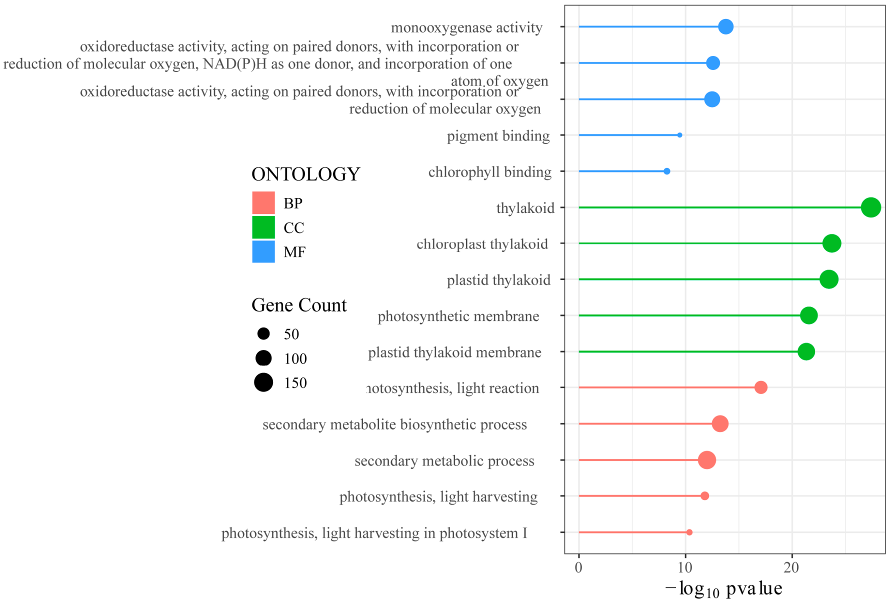

Figure S10: GO enrichment analysis of the down- regulated genes from S1 to S3.

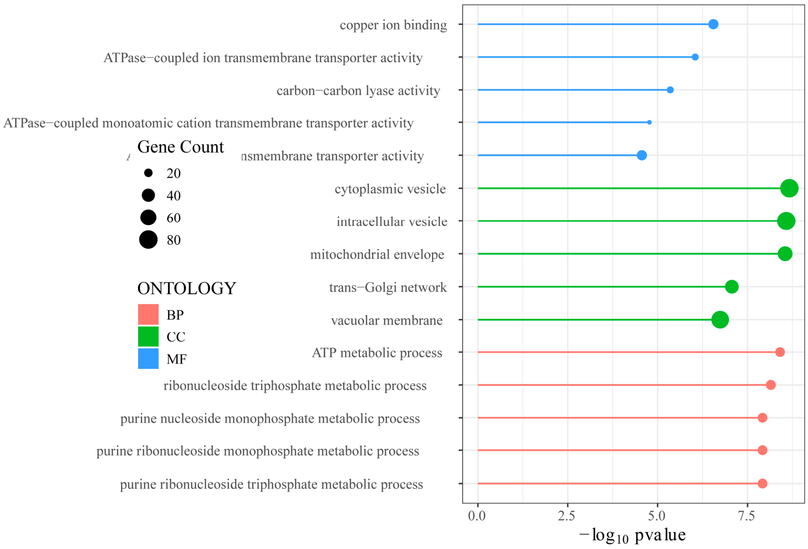

Figure S11: GO enrichment analysis of the up- regulated genes from S1 to S3.
